# Road Sign Recognition with Fuzzy Adaptive Pre-Processing Models

**DOI:** 10.3390/s120506415

**Published:** 2012-05-15

**Authors:** Chien-Chuan Lin, Ming-Shi Wang

**Affiliations:** Department of Engineering Science, National Cheng Kung University Taiwan, No.1, University Road, Tainan City 701, Taiwan; E-Mail: mswang@mail.ncku.edu.tw

**Keywords:** road sign recognition, fuzzy inference, Adaboost classifier, support vector machine

## Abstract

A road sign recognition system based on adaptive image pre-processing models using two fuzzy inference schemes has been proposed. The first fuzzy inference scheme is to check the changes of the light illumination and rich red color of a frame image by the checking areas. The other is to check the variance of vehicle's speed and angle of steering wheel to select an adaptive size and position of the detection area. The Adaboost classifier was employed to detect the road sign candidates from an image and the support vector machine technique was employed to recognize the content of the road sign candidates. The prohibitory and warning road traffic signs are the processing targets in this research. The detection rate in the detection phase is 97.42%. In the recognition phase, the recognition rate is 93.04%. The total accuracy rate of the system is 92.47%. For video sequences, the best accuracy rate is 90.54%, and the average accuracy rate is 80.17%. The average computing time is 51.86 milliseconds per frame. The proposed system can not only overcome low illumination and rich red color around the road sign problems but also offer high detection rates and high computing performance.

## 1. Introduction

The term Intelligent Transportation System (ITS) refers to approaches that attempt to integrate advanced information, electronic, control, sensor, and communication technologies into both the transport infrastructure and vehicles in order to better manage transportation. The ITS provides the tools for skilled transportation professionals to collect and react to data about the performance of a system, and one of primary benefits of such systems is that much of the data is real time. Having this real time data enhances drivers' ability to respond to incidents, adverse weather or other capacity constricting events. The main objective of an ITS is to improve transportation safety and reduce vehicle wear, transportation time, and fuel consumption. ITS encompasses a broad range of wireless and wired communications-based information and electronics technologies. The driver assistance systems (DAS) are one of the important aspects of ITS, as a well-designed human-machine interface should be able to increase individual car safety and road safety in general. Examples of DAS include in-vehicle navigation systems, lane change assistance, automatic parking, driver drowsiness detection, and traffic sign recognition.

Road traffic sign detection and recognition system is one of vehicular assistance system employed for the recognition of stop signs, speed limits, and prohibitory, cautionary and guidance signs. The main problems in road traffic sign detection and recognition are that the signs may be viewed from odd angles, under low illumination, and partially occluded, and the rich red color that usually rings road signs also presents some challenges. In this paper, a vision-based road traffic sign recognition system was designed to process the mandatory and warning road signs used in Taiwan. The mandatory signs have a red circular ring and white background symbol(s) within the circle. The warning signs are have a red triangular rim, with a white background and black symbol(s) within the triangle.

The main modules of the proposed system are a fuzzy image checking model scheme to perform image pre-processing, an adaptive boosting classifier to detect the candidate road signs and a support vector machine to recognize them. The image pre-processing phase is very important, especially in road sign detection, because the road environment is extremely variable. Consequently, fuzzy color enhancement [1,2] and contrast enhancement [3] are both applied in our system.

## 2. Related Works

Freund and Schapire [4] proposed their adaptive boosting (Adaboost) algorithm in 1997, and this is a machine learning method to decrease errors in exponentiation. Viola and Jones [5] used multiple weak classifiers in a cascade, and gave each classifier a weight to make a strong classifier. Using this approach, many strong classifiers can be combined in a cascade mode to implement the Adaboost algorithm. The Adaboost algorithm can retrain the last recorded classification errors to increase the detection accuracy. In practice, the Adaboost algorithm is used to combine multiple strong classifiers into a cascade mode scheme to classify a data set. Baro [6] proposed an Adaboost detection and forest-ECOC (error-correcting output code) classification approach to recognized traffic sign with high performance and accuracy. Baro's method is based on embedding a forest of optimal tree structures in the ECOC framework.

In 1995, Vapnik [7] proposed a machine learning theory, the called support vector machine (SVM) method, derived from statistic learning theory, and this has become one of the most popular classification schemes, and has been adapted and applied in many different domains. The SVM method is a high performance classifier that has been extended to classify both linear separable and linear inseparable data sets. In practical applications, most data sets are linear inseparable. Under this condition, the SVM uses the slack variable to overcome the low degree of data overlap, and the kernel function to deal with a high degree of data overlap.

Various methods for road traffic sign recognition have been proposed, and these are usually composed of two parts. The first is road sign detection which used to detect the road signs within the image, while the second is road sign recognition, which is used to understand the content of the road sign thus detected. Two cameras were employed in the system proposed by Miura [8], one equipped with a wide-angle lens (called a wide-camera) and the other with a telephoto lens (called a tele-camera). The wide-camera is used to detect objects, and the tele-camera is used to extract targets from the input image. The targets in their proposed system were circular signs and guidance signs, and Hough transformation was applied to detection of the former. However, if a road sign is not perpendicular to the camera lens, then this detection scheme is likely to fail. Gao [9] proposed a system in which the input images first had to be transformed into the CIECAM97 color space so that regions of interest could be extracted. This system used to extract the shape features, with 49 sensor nodes obtained. Finally, the density and the edge orientation of these 49 areas can be as templates, with a matching scheme used to recognize the road signs. Liu [10] employed color equalization in a HSV color space to separate the color information in road signs. The area and aspect ratio were used to detect the Regions of Interest (ROI), which were then normalized to obtain 32 distributed radial areas. To obtain the final results, the histogram of the 32 areas was calculated and then matched with templates in the database. However, this approach is not very successful if road signs are under low illumination or partially occluded. Saturnino [11] proposed a road sign recognition system based on an RBF-kernel support vector machine, in which the HSI color information is used to extract the road sign candidates, and the shape of the road signs are recognized by using a linear support vector machine. Their approach, however, detected too many candidates in the detection phase, and thus required much more computing time to process these. Shi [12] focused on recognizing seven categories of road sign shapes and five categories of speed limit signs. Two kinds of features, binary image and Zernike moments, were used for representing the data to the SVM for training and testing. They found that the SVM produced better results using the binary image rather than the Zernike moments. Koncar [13] designed a hierarchical road sign classifier, and in their approach the Gabor wavelet transform was used to produce a Gabor similarity map, and the recognition process was similar to a decision tree by template matching. Barnes [14] proposed a system with a radial symmetry detector to detect circular signs more rapidly. Because the gradient orientations of the pixels that belong to the circumference are oriented to the center of the circle, this property was employed to aid the process. Their results showed that the detection time with this system was less than with the Hough transform method. Yoon [15] applied fuzzy measures and fuzzy integrals to extend the Adaboost algorithm, in a process called fuzzy-boosting, and the classifier produced by this approach was then used to detect road guidance signs.

Most of the above related works emphasized the achievement of a high level of accuracy. However, the highly variable environment of real roads was not discussed in detail. For example, road signs in any captured image are likely to suffer from low illumination, and blend with the background. Under these conditions, the correct detection rate will be decreased. The efficiency of a system is another important issue. In order to solve the above problems, we propose a system in this paper that not only robustly detects the road signs correctly for the images under variable and adverse conditions, but also works efficiently.

## 3. The Proposed Road Sign Recognition System

The proposed road sign recognition approaches are divided into three phases, which fuzzy adaptive models employed to pre-processing phases, road sign detection using Adaboost classifiers scheme and road sign recognition using SVM classifiers technique.

### 3.1. Fuzzy Adaptive Checking Area

Both prohibitory and warning signs in Taiwan have red rims. In order to detect road signs more quickly, red information will be found first when an image is input into the system, and the hue, saturation and intensity (HSI) color space is used to achieve this. Due to variations in the environment, the brightness of the captured images also varies:

(1) $$\text{RedPixel}=\{\begin{array}{ll} H\geq h_{1} & \text{or}\;H\leq h_{2}\\ S>a & \\ I>b & \end{array}$$

According to our experiments, giving sufficient values of the threshold conditions shown in Equation (1) could enable the system to find the appropriate number of the red pixels in a road sign, and get good results in most illumination environments. The above detection scheme, however, may fail with images taken under the following three conditions: against the sunlight (e.g., against the sky); with a darker background (e.g., low illumination), and in a scene with a lot of red. From observation of the real data, in most cases, the saturation of red color in a road sign is greater than the surrounding red background. Therefore, one way to address this case is to increase the threshold value of the saturation component. However, different situations may be combined together to reduce the performance of the system correction rate.

**Figure 1.**
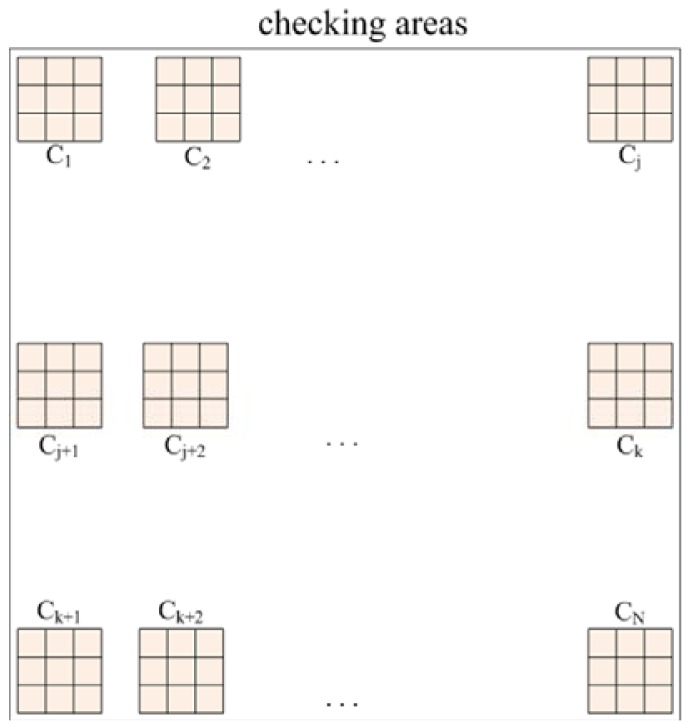
The N checking areas, each with a 3 × 3 pixels block, which are allocated at the upper edge, the middle portion, and the bottom edge of the image, where j = N/3 and k = (2N)/3.

Consequently, a scheme known as fuzzy checking areas is proposed, which first checks the illumination and red color component of the input image, and then the image is classified according to the results of these evaluations and the appropriate further processing procedure is selected. Assume N checking areas, each with a 3 × 3 pixels block, are located along the upper edge (called upper checking row), the middle portion (called middle checking row), and the bottom edge (called bottom checking row) of the image, each with N/3 checking areas and distributed uniformly within the checking row, as shown in Figure 1.

The intensities and red color components of these checking areas are used, via the fuzzy logic inference method, to evaluate the image condition. After the fuzzy logic inference operation, the image will be classified into one of six classes: normal, situation 1 (bright background), situation 2 (too dark), situation 3 (rich red color), situation 1 and 3 (bright background and rich red color), or situations 2 and 3 (too dark and rich red color) as shown on the column named situation of Table 1

To evaluate the intensity level of an image, a fuzzy member function is defined and estimated from the image. Assume CI_i_ denotes the average intensity of the i^th^ checking area, normalized to the closed interval of 0 and 1 as Equation (2). The w_i_ denotes the weighting value of the i^th^ checking area, and the checking areas located on the same checking row are given the same weighting value. The summation of all the w_i_ is equal to 1:

(2) $$CI_{i}=b/255$$

(3) $$F_{\mu }=\sum_{i=1}^{N}w_{i}CI_{i},\text{where}\{\begin{array}{l} w_{i}=\frac{P_{1}*3}{N}if1\leq i\leq j\\ w_{i}=\frac{P_{2}*3}{N}if\;j<i\leq k\\ w_{i}=\frac{P_{3}*3}{N}if\;k<i\leq N \end{array}$$

With regard to real world environments, for intensity checking, the weight ratios of these checking areas located on the upper, middle, and bottom checking row are set at P_1_, P_2_, and P_3_, respectively. The sum of P_1_, P_2_, and P_3_ equals 1. This means that if there are N checking areas, the weights for each area of the upper, the middle, and the bottom checking rows are P_1_ /(N/3) = P_1_*3/N, P_2_ /(N/3) = P_2_*3/N, and P_3_ /(N/3) = P_3_*3/N, respectively. Let F_μ_ be the weighted intensity summation of the N checking areas, as shown in Equation (3), then F_μ_ is used as the fuzzy member function to evaluate the intensity of the input image as either dark, normal, or bright.

To check that if the whole input image is rich in red, a fuzzy member function to evaluate the red in the image is defined and estimated from the image. The CR_i_ is used to denote if the average red color component is greater than both the blue and green color components of the i^th^ checking area. The CR_i_ is set to 1 if (R_i_ − G_i_) > 0 and (R_i_ − B_i_) > 0. Otherwise, it is set to 0, where R_i_, G_i_ and B_i_ are the average red, green, and blue color values of the i^th^ checking area, respectively. To check the intensity of the red, each checking area is also assigned a weighting value and all the checking areas located on the same checking row have the same weights. The distribution of the total weights for the upper, middle, and bottom checking rows are thus given to P_4_, P_5_, and P_6_, respectively. The sum of P_4_, P_5_, and P_6_ equals to 1. This means that if there are N checking areas, the weighting values for each area of the upper, middle, and bottom checking rows are P_4_ /(N/3) = P_4_*3/N, P_5_ /(N/3) = P_5_*3/N, and P_6_ /(N/3) = P_6_*3/N, respectively:

(4) $$CR_{i}=\{\begin{array}{lll} 1ifR_{i}-G_{i}>0 & \text{and} & R_{i}-B_{i}>0\\ 0 & & \text{otherwise} \end{array}$$

**Figure 2.**
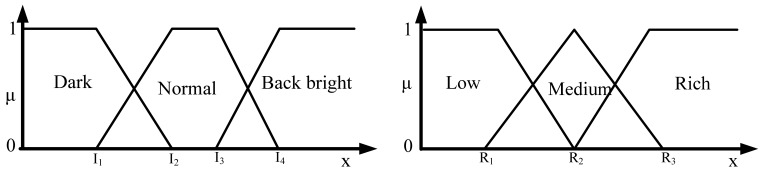
The membership functions of illumination and the membership functions of red.

**Figure 3.**
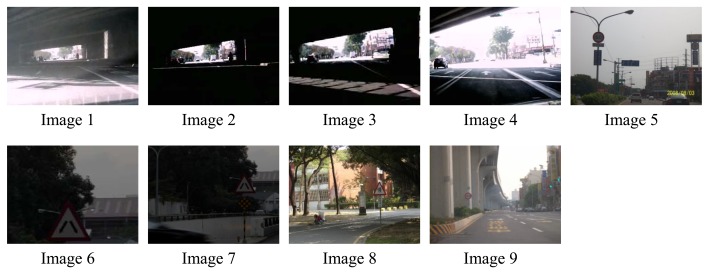
The nine testing images for fuzzy checking area detection.

**Figure 4.**
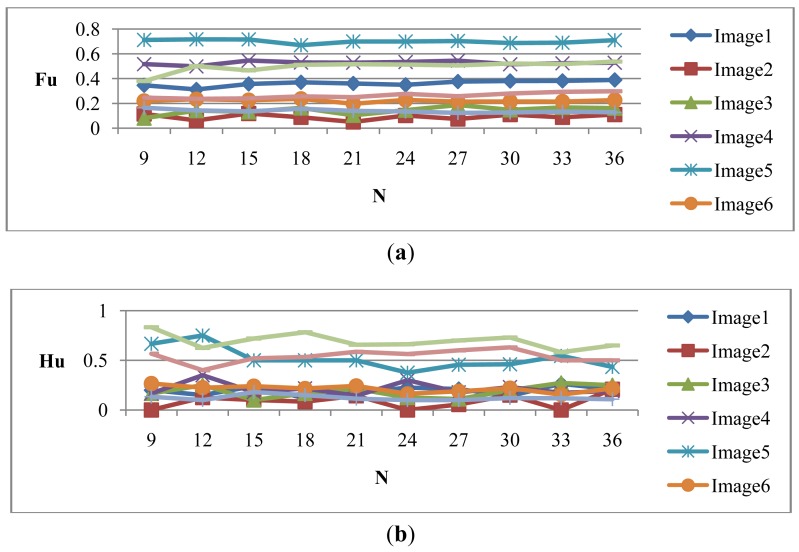
(**a**) The F_μ_ value curves and (**b**) the H_μ_ value curves of the nine testing images with different numbers of checking areas for Figure 3.

Let H_μ_ be the weighted rich red color summation of the N checking areas, as Equation (4), where v_i_ denotes the weighting value of the i^th^ checking area, then H_μ_ is used as the fuzzy member function to evaluate the richness of the red color in the input image, as Equation (5). Figure 2 shows the relationship of the fuzzy member functions for illumination and red color, respectively. Figure 3 illustrates the testing data for the fuzzy adaptive checking area scheme. Figure 4 shows that the F_μ_ and H_μ_ values for the nine testing images in Figure 3, with different numbers of checking areas. A comparison of the images and the corresponding F_μ_ and H_μ_ values shows that the F_μ_ and H_μ_ values could correctly indicate the illumination and red color information of the images.

(5) $$H_{\mu }=\sum_{i=1}^{N}v_{i}CR_{i},\text{where}\{\begin{array}{l} v_{i}=\frac{P_{4}*3}{N}if1\leq i\leq j\\ v_{i}=\frac{P_{5}*3}{N}if\;j\leq i\leq k\\ v_{i}=\frac{P_{6}*3}{N}if\;k\leq i\leq N \end{array}$$

Table 1 lists the fuzzy rules and their results for the inferences. According to these rules, the input image can be classified as belonging to one of nine situations. If the image is classified as a normal one, the threshold condition shown in Equation (1) with h_1_ = 330, h_2_ = 30, a = 0.2 and b = 30 is applied to obtain the red information for road sign detection.

If the image is evaluated as belonging to situations 1 or 2, then the image is first contrast enhanced and then the threshold condition shown in Equation (1) with h_1_ = 345, h_2_ = 15, a = 0.4 and b = 30 is employed. If the image is rich in red color, which may occur with situations 1 or 2, as shown in Table 1, then contrast enhancement is applied to the image and the threshold condition would be change as h_1_ = 345, h_2_ = 15, a = 0.4 and b = 30 that is applied to the HSI image of the enhanced version.

### 3.2. Fuzzy Detection Area for Video Frames

**Figure 5.**
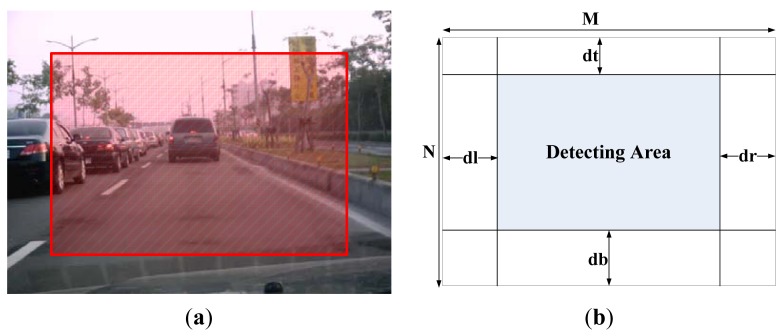
(**a**) The field of driver's view (FODV); (**b**) The detecting area and discarded portions.

We employ fuzzy technology to obtain an adaptive region of interesting. Figure 5(a) shows an example of a captured image from the driver's viewpoint. It was taken from a camera installed in front of the driver's seat in a car. Of course, this view is related to where the camera is installed, and the field of driver's view (FODV) in a video frame is fixed when the camera is fixed in the car. Most of the content is the same in two adjacent frames. Usually the areas in the lower, right, and left portions of an image cannot provide new information, because the data contained in these areas is also in the previous frames. According the experimental results, the portion located in the top area of the image contains either the skyline or road signs that are too small to be detected. Therefore, for video sequence processing, the detecting area (DA) of a frame image is reduced from the original one to increase the processing speed. The area for detecting new objects in the current frame is presented at the top portion of the previous frames.

Figure 5 shows the relationship between FODV and DA. The clipped parameters ***dr, dl, dt***, and ***db*** shown in Figure 5(b) can be adapted for applications which are dependent on the location of installed video camera, the driving direction of the car, and the status of road, for example, turning right, turning left, on an upgrade and on a downgrade. When the DA is larger, the processing time is longer and more objects can be detected.

**Figure 6.**
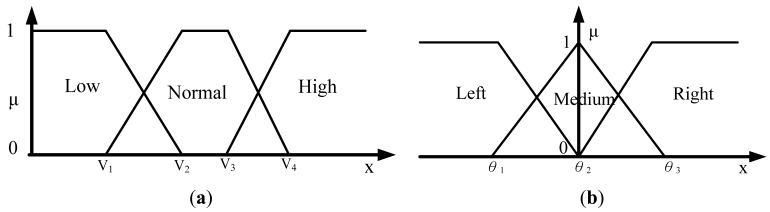
Membership functions of vehicle speed, and membership functions of the angle of the steering wheel. (**a**) The fuzzy member functions of speed **V.** (**b**) The fuzzy member functions of steering wheel ***θ***.

To find these adaptive parameters, fuzzy inference technology is adopted according to different vehicle driving environments. Suppose the speed and angle of the steering wheel of the vehicle system can be provided. Then parameters ***dt, db, dr***, and ***dl*** can be used as adaptive parameters of the vehicle driving status. Assume that the vehicle driving direction is roughly classified as forward, turning left or turning right, the vehicle speed is roughly classified as low, normal or high. The fuzzy member functions of speed **V** and the angle of the steering wheel ***θ*** can be obtained by Figure 6(a,b), respectively, where the turning right angle is positive and the turning left angle is negative.

Table 2 illustrates the rules of the fuzzy logic system to obtain the adaptive size and position of the DA. There are three DA cases for when the vehicle driving status is at low, normal or high speed with the medium angle of steering wheel.

**Figure 7.**
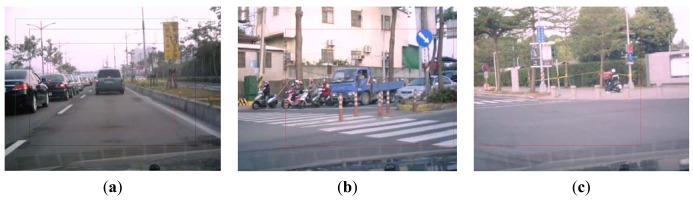
(**a**) Adaptive DA with different speeds: the middle rectangle for normal speed, the inner one for high speed, and the outer one for slow speed; (**b**) adaptive DA examples: turning right; (**c**) adaptive DA examples: turning left.

The outer rectangle area is the DA for low speed, the middle rectangle area is for normal speed, and the inner rectangle area is for high speed. Figure 7(a) shows that three DA cases when the vehicle driving status is at low, normal or high speed with the medium angle of steering wheel. The outer rectangle area DA is at low speed; the middle rectangle area is at normal speed, and the inner rectangle area is at high speed. Figure 7(b,c) show two cases for adapting the DA to the vehicle driving status while it is turning right and turning left with normal speed, respectively. In Figure 7(b), the position of DA has been moved to the right, and to the left in Figure 7(c).

### 3.3. Road Sign Detection Phase

After the preprocessing described in the previous subsection, only the red pixels are left in the detecting area of the image. The road sign detection phase evaluates if there are any signs included within the red pixels detected in the image. The size and location of the road sign candidates are reported if the system finds any possible road sign candidates in this phase. Finally, the detected road sign candidates are segmented from the original image as the input for next stage of processing.

Two Adaboost classifiers are trained to detect road sign candidates in this study, one is for prohibitory signs, and the other is for warning ones. The aim of our schemes is to overcome such problems as low illumination, viewpoint rotation and partial occlusion. The training data for the Adaboost classifiers includes both positive and negative samples. The negative training samples mean that no desired road signs are contained in the image, while the positive training data all include a road sign. In this study, the positive samples for prohibitory signs are included in the negative samples for warning signs, and the positive samples for warning signs are also included in the negative samples for prohibitory signs.

### 3.4. Road Sign Recognition Phase

The segmented road sign candidates generated from the road sign detection phase are further processed by the road sign recognition module. The function of the road sign recognition phase is to decide if the candidates are truly road signs or not. If they are, then the specific one is determined, and so the main work of this phase is to recognize the content of the detected candidate road sign.

**Figure 8.**
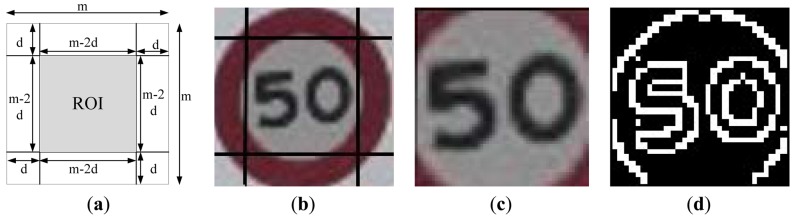
(**a**) The selected ROI for the extracted road sign candidate image; (**b**) its corresponding source image; (**c**) the ROI of (b); (**d**) the binary edge image of (a).

The input image data for this phase is the extracted road sign candidates obtained from the previous phase, which is a square image. The road sign recognition process is as follows: first, the ROI from the road sign candidate image is selected and normalized into a 30 × 30 pixel block to get a higher recognition rate. Second, the Canny edge detector is employed to obtain the contour information of the ROI and transform it into a binary edge image. Third, the feature vector of the ROI is formed from the binary edge image. The next step is to use the corresponding SVM, circular (prohibiting) or triangle (warning), to recognize the content of the ROI and then output the result. Assume the size of the detected road sign candidate image is *m* × *m* pixels and *d* is the number of pixel which would be cut out from each edge of the candidate image, then the size of the ROI will be (*m* − 2*d*) × (*m* − 2*d*). For this, the ROI will contain the content of the road sign only, as shown in Figure 8(a). The ROI is normalized into a 30 × 30 pixel block so that the images input into the classifier are all of a uniform size. In this approach, the contour information of the ROI is applied to represent its feature vector, and the Canny edge detector is used to obtain its contour information. For example, Figure 8(d) shows the binary edge image of Figure 8(b). In Figure 8(d), the pixel value for these edge pixels is set to 1 (white), while the others are set to 0.

**Figure 9.**
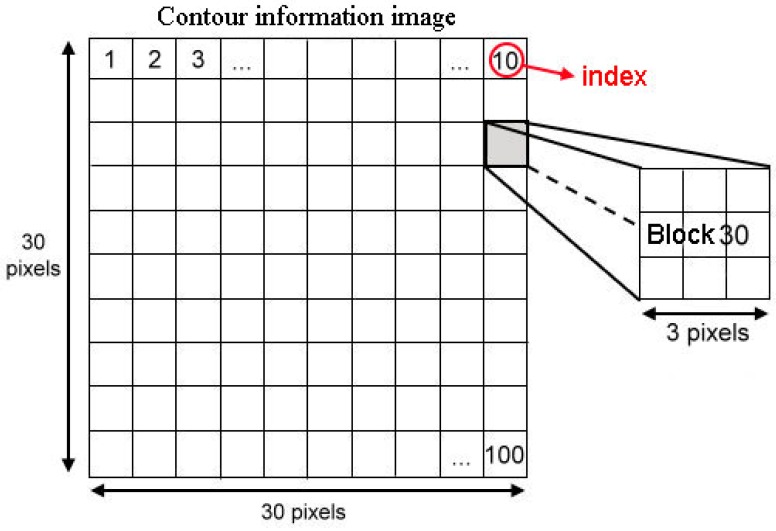
Reduction of dimensions of the feature vector.

To reduce the feature vector dimensions and reduce processing time, the binary edge image is divided into a 10 × 10 grid, as shown in Figure 9, to obtain a 100 components feature vector. The value of each component of the redesigned feature vector is set as the sum of the values of the corresponding 3 × 3 edge image block. This redesigned feature vector is used as the input data of the SVM classifier.

Two SVM classifiers are employed, one for prohibition signs and the other for warning signs to recognize the road sign contents. In our proposed system, the prohibitory road signs are divided into 34 classes and warning road signs are also divided into 34 classes. Besides total 68 classes of road signs, the “non-road-sign” class also included, too. In the proposed system, both the prohibitory and the warning sign SVM classifiers are designed to output 34 different classes of the corresponding road signs, and 30–45 training samples are provided for each class. For the “non-road-sign” class, 75 training samples are used for training in both of the SVM classifiers. The C-SVC scheme [16] is adopted in the proposed system. The kernel function of C-SVC is a radial basic function (RBF) and the parameters to be set are C and *γ*.

## 4. Experimental Results

The proposed system has been implemented in C++ language and developed on a PC platform with an Intel Core 2 Duo 2.4 GHz CPU and 2 GB RAM. The training data for each of the two Adaboost classifiers are 350 positive and 1,850 negative samples. The number of test images is 350 that included 242 prohibitory road signs, 223 warning road signs and 38 non-road signs, as listed in Table 3. The LIBSVM library [16] support vector machine was used in the proposed system. The parameters of SVM: for the prohibitory sign C = 4 and *γ* = 0.00390625, and for the warning sign C = 2 and *γ* = 0.00390625. The number of checking areas N = 9. The weight ratios of checking areas P_1_ = 0.5, P_2_ = 0.4, P_3_ = 0.1, P_4_ = 0.4, P_5_ = 0.5 and P_6_ = 0.1. Table 4 lists the training and testing data of SVM classifier.

### 4.1. The Results of Static Images

The image size is 320 × 240 pixels. The experiment has three aims as follows: (1) To check the effectiveness of the SVM classifiers; (2) To determine the minimum size of the road signs that could be detected under the image resolution used, and thus to estimate the distance that can be effectively processed by the proposed system; (3) To determine the average processing time for one image by adjusting the frame rate of the video sequence used in the second part of experiment. As not all of the testing images were taken from the field of driver's view, the whole testing image was processed as the detection area, meaning that the testing image was not reduced in size in this experiment.

**Figure 10.**
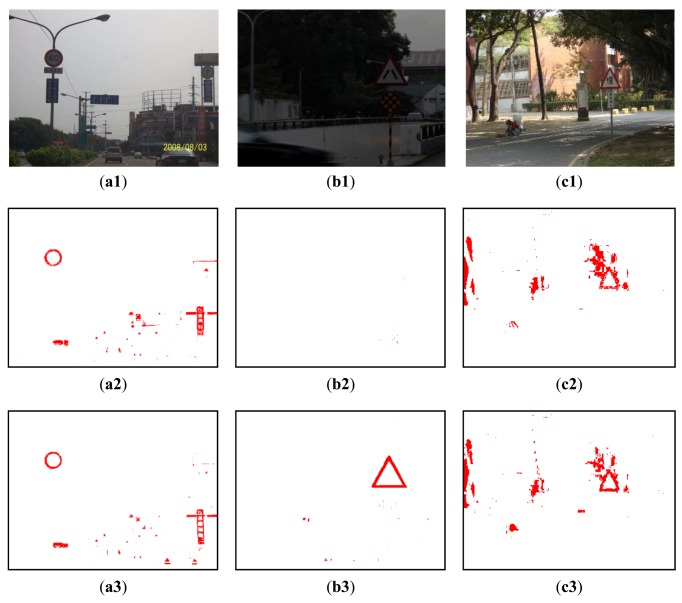

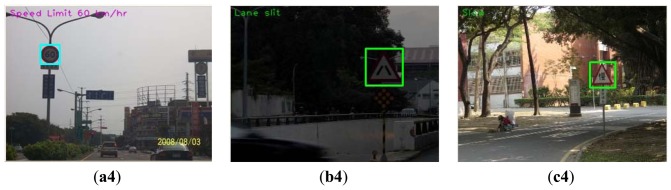
Three different situations considered: (**a1**) bright background; (**b1**) too dark; (**c1**) rich red color image; (**a2**)–(**c2**) the corresponding detection results of (**a1**)–(**c1**) using Equation (1) with h_1_ = 330, h_2_ = 30, a = 0.2 and b = 30; (**a3**)–(**c3**): the corresponding detection results of (**a1**)–(**c1**) using Equation (1) with h_1_ = 345, h_2_ = 15, a = 0.4 and b = 30. (**a4**)–(**c4**) are the recognition results.

The common detection scheme, however, may fail with image under the following three conditions: (1) against the sunlight (e.g., against the sky); (2) With a darker background (e.g., low illumination); (3) In a scene with a lot of red. Examples of these cases are shown in Figures 10(a1), (b1), and (c1), respectively. Figures 10(a2), (b2), and (c2) show the corresponding detection results produced using Equation (1) with h_1_ = 330, h_2_ = 30, a = 0.2 and b = 30 from Figures 10(a1), (b1), and (c1). It can be seen that they are not sufficient. In situation 1 (Figure 10(a1)) and situation 2 (Figure 10(b1)), the red pixels look like black ones, and most of the red pixels will be discarded after the threshold operations using Equation (1) with h_1_ = 345, h_2_ = 15, a = 0.4 and b = 30. This is due to the low saturation of the red, and so one way to solve this problem is to reduce the threshold of the saturation component. In situation 3, too many red pixels are retained. From observation of the real data, in most the cases the saturation of the red color of a road sign is greater than that of the surrounding red background and so one way to address this is to increase the threshold value of the saturation component. However, in real applications, different situations may be combined together to reduce the performance of the system correction rate.

A total of 350 test images, 242 prohibitory road signs and 223 warning road signs were included in these test images. After the road sign detection stage, 234 prohibitory road signs, 219 warning road signs and 38 non-road signs were detected. After analyzing the detection results, the minimum size of road sign which could be detected was 20 × 20 pixels, from which it is estimated that the effective distance for the proposed system is about 20 meters from the camera. The average processing time for one image was 118.7 milliseconds.

**Figure 11.**
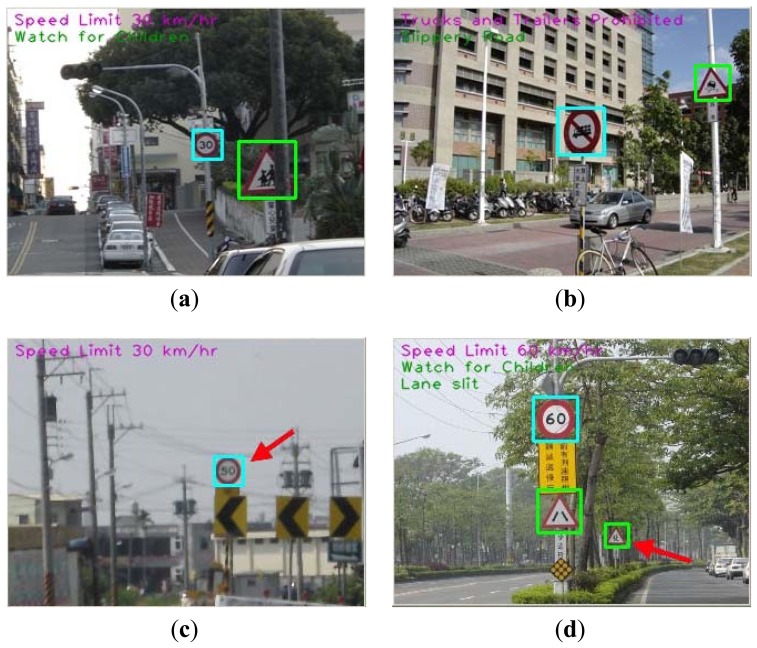
Four samples of experimental results. (**a**) and (**b**) are correct cases; (**c**) is a error case; (**d**) is two correct and one error case.

Figure 11 shows four samples of experimental results. The images (a) and (b) are correct cases with partially occluded road signs. The image (c) is an error case. In this case, the road sign has been detected, but the content of the road sign is not clear. The image (d) is two correct and one error case. The error reason is the location of road sign too far away, but we still detected it.

Tables 5 and 6 show the processing results of the static images. After the recognition stage, 219 prohibitory road signs, 211 warning road signs, and 38 non-road signs were recognized correctly. In the detection phase, the detection rates are 96.69% and 98.21% for prohibitory and warning road signs, respectively. The average detection rate is 97.42%. In the recognition phase, the recognition rates 91.79% and 95.40% for prohibitory and warning road signs. The average recognition rate is 93.04%. The system true positive rates are 90.50% and 94.62% for prohibitory and warning road signs. The average false negative rate is 0%. Finally, the average true positive rate is 92.47%.

Table 7 compares the average accuracy rate of different method with different recognition road sign type in the static image. The first five methods [12] focused on road sign shapes and speed limit signs classification. The best average accuracy rate is 99.5% (SVM & BI) and the worst average accuracy rate is 69.0% (FA & ZM & PCA). Adaboost & F-ECOC method [6] classified circular, triangular and speed road signs. The average accuracy rate is 94.0%. The proposed method focused on prohibitory and warning road signs. The proposed method not only classified road sign shapes, but also recognized the content of these road signs.

### 4.2. The Results of Video Sequences

The testing data included 10 video sequences which were recorded with the camera installed in front of the driver's seat. The image frame size was 320 × 240 pixels and the frame rate was 10 frames per second. Two video sequences were recorded on a cloudy day and the others were recorded on a sunny day. Because the information included in two adjacent image frames is almost the same, except for the depth of the view, usually the area of the lower, right, and left portions of a frame image are processed on the previous frames. In addition, based on the results in the previous subsection, the road signs located in the top portion of a frame image are too small to be detected. Therefore, for video sequences testing, the detecting area of a frame image is reduced to increase the processing speed.

**Figure 12.**
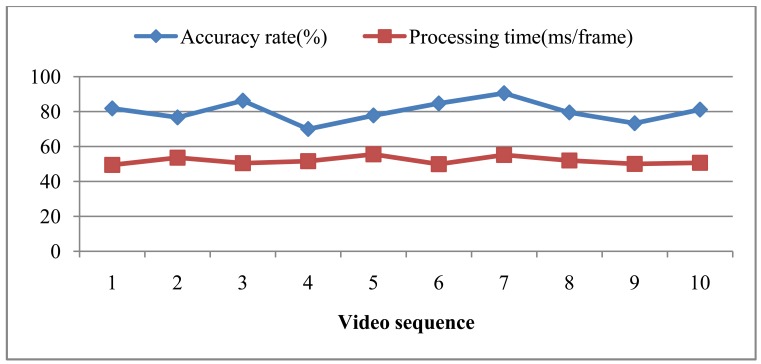
The experimental results for the 10 testing video sequences.

Figure 12 shows the results for these 10 testing video sequences that show the accuracy recognition rate and the average processing time per frame in each video sequence. The worst recognition rate is 70.0%, the best accuracy rate is 90.54%, and the average recognition rate is 80.17%. There is thus a large gap in recognition rate between the best and the worst rates. After inspecting the road conditions where the video sequences were taken, it was found that the road was too bumpy for recording video sequence #8, causing lower image quality. The total average computing time is 51.86 ms/frame. According to the average the computing time, the proposed system can handle 19 frames per second.

Table 8 compares the accuracy rate and average processing time between fuzzy boosting [15] and proposed methods in the video sequences. The average accuracy rate of fuzzy boosting is better than our proposed method. But the proposed best accuracy rate and the average processing time are better than fuzzy boosting method.

**Figure 13.**
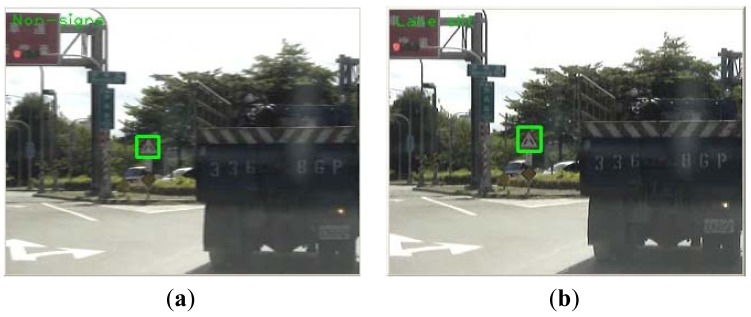
Blurred image may cause error: (**a**) the detection result of the 41st frame of video sequence #08; (**b**) the next frame, which can be recognized correctly.

In addition, the recorded images tend to blur when the car swings left or right, also causing errors in detection and recognition. Figure 13(a) is the 41st frame of video sequence #08, and shows that no sign was detected, and this is because the vehicle started to turn right at that moment. We conjecture that this problem is related to the format of the source video sequence file. When the vehicle encounters vertical variation (vibration) or horizontal variation (turning left or right), it causes mistakes in the prediction of the motion vector and thus and causes the frame to be blurred. However, usually the adjacent frame could be recognized correctly, as Figure 13(b) shows, which successfully detected a road forking sign.

**Figure 14.**
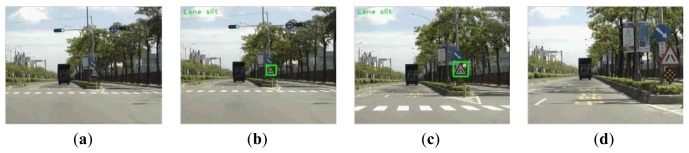
Road sign appearing in a sequence of frames: (**a**) too small to be detected; (**b**) correctly recognized; (**c**) correctly recognized; (**d**) out of the detecting area and thus no detected.

Figure 14 shows some example frames in a sequence. According to the detecting area setting, Figure 14(a) shows the case that the road sign is in the detecting area but too small to detect. The road sign in Figure 14(b,c) can be detected and correctly recognized. Figure 14(d) shows the target is out of the detection area, and thus the system cannot detect it.

In practice on vehicles, the performance is an important issue. The experimental results show that the proposed scheme not only provides high detection and recognition rate, but also give a high computing performance. The performance of the average processing time is excellent. The proposed system could process video sequence above 15 frames per second. Thus the proposed system is suitable for a real road sign recognition application in the vehicle.

## 4. Conclusions

In this paper, an adaptive image pre-processing road sign recognition system has been proposed, which employed fuzzy adaptive image checking areas and fuzzy adaptive detection area selection. The Adaboost classifier to detect candidate road signs and support vector machines to recognize the content of the detected candidate road signs are used. The proposed system can not only overcome low illumination and rich red color around the road sign problems but also offers a high detection rate and high computing performance. Although the sample data are about prohibitory and warning road traffic signs, however, it is easy to extend these approaches to others using similar technology.

## Figures and Tables

**Table 1.** Fuzzy rules for determining image situations.

**Input**		**Output**	

**Illumination**	**Red Color Area**	**CF (Certainty Factors)**	**Situations**
Dark	Low	Dark-Low	S2
Normal	Low	Normal-Low	Normal
Bright	Low	Bright-Low	S1
Dark	Medium	Dark-Medium	S2
Normal	Medium	Normal-Medium	Normal
Bright	Medium	Bright-Medium	S1
Dark	Rich	Dark-Rich	S2 & S3
Normal	Rich	Normal-Rich	S3
Bright	Rich	Bright-Rich	S1 & S3

**Table 2.** The rules of the fuzzy logic system for the adaptive DA.

**Input**		**Output**

**Size of DA**	**Position of DA**	**CF (Certainty Factors)**
Low	Left	Low-Left
Low	Medium	Low-Medium
Low	Right	Low-Right
Normal	Left	Normal-Left
Normal	Medium	Normal-Medium
Normal	Right	Normal-Right
High	Left	High-Left
High	Medium	High-Medium
High	Right	High-Right

**Table 3.** The Adaboost classifier training and testing data.

**Dataset**	**#Training**		**#Testing**	

	**Prohibitory**	**Warning**	**Prohibitory**	**Warning**
#Positive	350	350	242	223
#Negative	1,850	1,850	38	38

**Table 4.** The SVM classifier training and testing data.

**Dataset**	**#Training**		**#Testing**	

	**Prohibitory**	**Warning**	**Prohibitory**	**Warning**
#Class	34	34	34	34
#Sample	1,212	1,235	234	219
#Non-Road Sign	75	75	38	38

**Table 5.** The experimental results of standard metrics for static images.

**Confusion Matrix (Standard Metrics)**		**Predicted Sign**		**Predicted Sign**		**Total**	
		**Prohibitory**	**Non- Prohibitory**	**Warning**	**Non-Warning**	**Road Sign**	**Non-Road Sign**
Actual Sign	Road Sign	219	0	211	0	430	0
	Non-Road Sign	23	38	12	38	35	38

**Table 6.** The experimental results of detection and recognition for static images.

**Item**	**Prohibitory**	**Warning**	**Total**
Detection Rate	96.69%	98.21%	97.42%
Recognition Rate	91.79%	95.40%	93.04%
False Negative Rate	0%	0%	0%
True Positive Rate	90.50%	94.62%	92.47%

**Table 7.** The comparison with other methods for accuracy rate in static image.

**Method**	**Average Accuracy rate**	**Recognition type**
FA & ZM	86.3%	Road sign shapes & Speed limit signs
FA & ZM & PCA	69.0%	Road sign shapes & Speed limit signs
FA & ZM & LDA	98.0%	Road sign shapes & Speed limit signs
SVM & ZM	91.7%	Road sign shapes & Speed limit signs
SVM & BI	99.5%	Road sign shapes & Speed limit signs
Adaboost & F-ECOC	94.0%	Circular, Triangular and Speed road signs
Proposed	93.0%	Prohibitory and Warning road signs

FA & ZM is the Fuzzy ARTMAP neural network with Zernike moments. SVM & BI is the SVM with binary representation.

**Table 8.** The comparison with other methods in video sequences.

**Method**	**Best Accuracy Rate**	**Worst Accuracy Rate**	**Average Accuracy Rate**	**Average Processing Time(ms)**
Fuzzy Boosting	87.0%	81.0%	84.67%	58.9
Proposed	90.5%	70.0%	80.17%	51.9
